# A Double Dissociation in the Roles of Serotonin and Mood in Healthy Subjects

**DOI:** 10.1016/j.biopsych.2008.10.001

**Published:** 2009-01-01

**Authors:** Oliver J. Robinson, Barbara J. Sahakian

**Affiliations:** Department of Psychiatry and Behavioural and Clinical Neuroscience Institute, University of Cambridge, Cambridge, United Kingdom

**Keywords:** Affect, cognition, depression, mania, mood, serotonin

## Abstract

**Background:**

Affective disorders are associated with altered cognitive performance. However, the precise interaction between affect and cognition is unclear. The manipulation of serotonin (5-HT), a neurotransmitter implicated in affect, influences performance on “hot” cognitive tasks that require the processing of affective stimuli, but manipulation of affect via mood induction influences performance on “cold” cognitive tasks that do not involve affective stimuli. We attempted to disentangle the influence of affect on cognition by examining the effect of manipulating both serotonin (via acute tryptophan depletion [ATD]) and mood on established hot and cold cognitive tasks.

**Methods:**

In a double blind, placebo-controlled crossover design, 33 healthy mood-induced (positive, negative, or neutral) subjects completed the (hot) affective go/no-go (AGNG) and (cold) one touch tower (OTT) following both placebo and ATD.

**Results:**

Mood influenced performance on the OTT but not AGNG; ATD influenced performance on the AGNG but not OTT.

**Conclusions:**

A double dissociation was demonstrated between the influence of ATD and mood on cognition, indicating that serotonin and mood are not closely linked. We hypothesize that this is due to the differences between emotions and moods and that aberrant cognition in affective disorders may be provoked through both bottom-up and top-down mechanisms.

Affect and cognition are dissociable, but interacting, mental processes (1,2). Correspondingly, affective disorders, such as depression and mania, are associated with changes in cognitive performance that can help to maintain the disease state (3). Understanding the interaction between affect and cognition is therefore crucial to a full understanding of affective disorders.

Cognitive processes can be divided into “cold” processes that are purportedly independent of affect and “hot” processes that require the processing of affective information (4). Consequently, experimental manipulations of neurotransmitter systems implicated in affective processing, such as serotonin (5-HT) (5), alter performance on “hot” cognitive tasks (6,7), while leaving performance on “cold” cognitive tasks intact (6). However, manipulation of affect via other methods, such as mood induction, can influence performance on the same “cold” tasks (1,8).

Affect therefore influences cognition beyond simply the processing of affective information. One reason for this is that affect is a multidimensional process that can be broken down into (at least) emotions, which are typically short-lived affective states triggered by specific stimuli and associated with specific autonomic changes; and moods, which are long-term background affective states with cumulative or unclear causes (1). Emotions often originate in midbrain and lower corticolimbic regions (9), whereas mood states are often subserved by prefrontal cortical regions (1,10,11).

This distinction may therefore help to clarify the role of 5-HT and mood in cognition. If 5-HT primarily alters emotional processing, it will influence tasks that require the fast processing of affective stimuli and may therefore influence “hot” tasks in a bottom-up fashion (5,6,12). If mood induction influences processing within prefrontal regions (such as those recruited by the Tower of London task [13,14]), it may influence performance on “cold” (but executive) cognitive tasks via a more top-down mechanism (9).

We therefore tested this hypothesis by manipulating 5-HT (via acute tryptophan depletion [ATD]) in three groups of individuals undergoing positive, negative, or neutral mood induction. All subjects completed the “hot” affective go/no-go (AGNG), which is influenced by ATD (6), and the “cold” one touch tower (OTT), which is not influenced by ATD (6). We predicted that ATD would influence the AGNG (via a bottom-up mechanism) and that mood would influence the OTT (via a top-down mechanism).

## Methods and Materials

### Experimental Procedure

Procedures were approved by the Norfolk Research Ethical Committee (06/Q0101/5). Thirty-four subjects (18 female subjects) were screened for psychiatric and neurological disorders (Table 1, Supplement 1). One subject did not complete either session, two subjects completed a single session, and one subject experienced technical difficulties with the AGNG. Subjects were assigned, double-blind, to the ATD-first group (*n* = 15) or nutritionally balanced (BAL)-first group (*n* = 19). Subjects were assigned to negative (*n* = 12, 4 female subjects), neutral (*n* = 10, 6 female subjects), and positive mood groups (*n* = 11, 7 female subjects) and tested on two sessions separated by at least 1 week. They were asked to consume only water from midnight prior to each session. At T_0_, a blood sample was taken and a nutritionally balanced (BAL) or a tryptophan free (ATD) amino acid drink was ingested. After approximately 5 hours, a second blood sample was taken (T_1_).

Subjects then completed mood induction procedures (MIP), followed by the affective go/no-go and then one touch tower tasks. Visual analogue scales (VAS) were completed to determine self-reported mood state. Further details are included in Supplement 1.

### Data Analysis

All data were analyzed via repeated-measures analysis of variance (ANOVA) in SPSS 10 (SPSS Inc, Chicago, Illinois). Error rates were square-root transformed. Simple effects were calculated from the estimated marginal means.

## Results

### Blood Sample Analysis

There was a significant two-way drink × time interaction for the critical tryptophan (TRP)/total long neutral amino acids (∑LNAA) ratio [drink × time: *F*(1,23) = 71.2, *p* < .0001]. Simple effects analysis revealed that this was due to a 84.7% decrease in the TRP/∑LNAA ratio between T_0_ and T_1_ in the ATD group [simple effect of time: *t*(23) = 12.2, *p* < .0001] but a 21.7% increase in the TRP/∑NAA ratio between T_0_ and T_1_ in the BAL group [simple effect of time: *t*(23) = −2.9, *p* = .007].

### Self-Report Mood

There was no treatment by time [*F*(1,29) = 1.4, *p* = .25] interaction between T_0_ and T_1_ on the happy - sad VAS, but there was a time by MIP interaction between T_1_ and T_2_ before and after the MIP [*F*(2,25) = 3.8, *p* = .035]. This demonstrates that the mood induction, but not ATD, successfully altered subjects' moods. Simple effects analysis is presented in Supplement 1.

### Affective Go/No-Go

**Figure 1:**
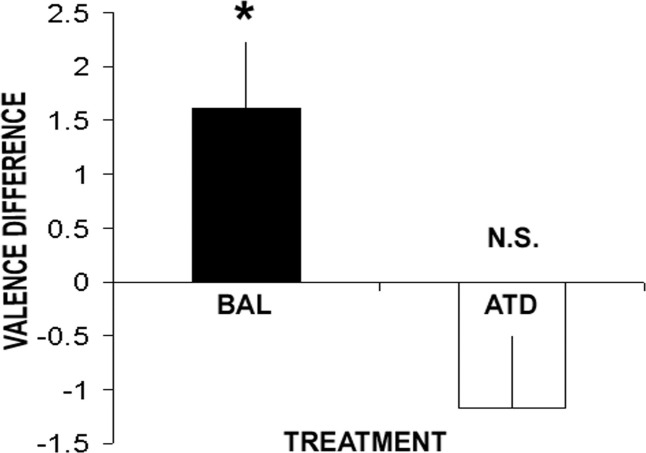
Performance on the “hot” AGNG is influenced by serotonin but not mood. Female subjects make significantly more errors in response to happy distracters than sad distracters (valence difference = happy - sad) under placebo (BAL). This bias is abolished by acute tryptophan depletion (ATD). * *p* < .05. AGNG, affective go/no-go; ATD, acute tryptophan depletion; BAL, nutritionally balanced; N.S., not significant.

There was no interaction between mood state, treatment, and word valance in the distracter (no-go) errors [*n* = 32, *F*(2,28) = 1.3, *p* = .30] or between mood state and word valence [*F*(2,28) = .62, *p* = .55]. However, there was a significant interaction between gender, treatment, and word valence [*n* = 32, *F*(1,29) = 8.8, *p* = .006]. Simple effects revealed an interaction between treatment and word valence in female [*n* = 16, *F*(1,14) = 7.2, *p* = .018] but not male subjects [*n* = 16, *F*(1,15) = 1.7, *p* = .21]. This female specific effect was driven by a significant increase in errors in response to happy distracter words (i.e, a positive bias) on placebo [main effect of valence: *F*(1,14) = 8.3, *p* = .009] but not following ATD [*F*(1,15) = 2.4, *p* = .14]. (Figure 1, Table 2, Supplement 1).

### One Touch Tower of London

**Figure 2:**
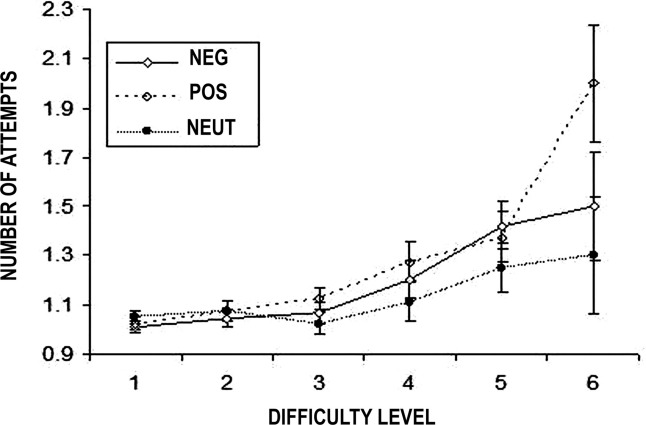
The number of attempts required to complete the “cold” one touch tower planning task is mediated by mood state, but not the serotonin: subjects in either a positive or negative mood make significantly more mistakes on harder problems. NEG, negative mood; NEUT, neutral mood; POS, positive mood.

There was a significant interaction between mood state and difficulty on the number of attempts required to complete problems [MIP × difficulty: *n* = 33, *F*(10,50) = 2.3, *p* = .017], which was not confounded by treatment [treatment × difficulty: *F*(5,24) = .49, *p* = .78] or by a treatment × mood interaction [treatment × mood × difficulty: *F*(10,50) = .67, *p* = .75]. Simple effects analysis revealed that this was due to a significant increase in the number of attempts required to complete the harder problems in subjects under negative [main effect of difficulty: *n* = 12, *F*(5,24) = 3.62, *p* = .014] or positive mood [*n* = 11, *F*(5,24) = 3.68, *p* = .013] but not neutral mood [*n* = 10, *F*(5,24) = 1.71, *p* = .17] (Figure 2, Supplement 1).

## Discussion

We demonstrate a double dissociation in the influence of serotonin and mood on cognition. The affective go/no-go task was mediated by serotonin but not mood manipulation, whereas performance on the one touch tower task was mediated by mood but not serotonin manipulation.

This finding is, to the best of our knowledge, the first experimental evidence that serotonin and mood are not closely linked. A recent meta-analysis (15) found no effect of serotonin manipulation on the mood state of healthy individuals. However, the reviewed studies largely relied on self-report of mood following 5-HT manipulation. Here, by manipulating both and by demonstrating a double dissociation in their influence over cognition, we indicate that 5-HT and mood state cannot be closely linked. This is of clear importance to our understanding of serotonergic function and its role in affective disorders.

The finding that 5-HT mediates performance on the AGNG has been demonstrated before (6) and indicates that reduced 5-HT, rather than negative mood, causes the disruption of AGNG performance found in depression (16). The restriction of this finding to female subjects is redolent of previous findings (15,17) and suggests that women are more susceptible to the effects of 5-HT fluctuation than men. This, in turn, may underlie the increased incidence (2:1) of depression in women.

The second finding, that mood state mediates performance on the OTT, replicates findings from the original Tower of London task (8,18) and demonstrates that both positive and negative mood can impair planning ability. Mood state, rather than altered serotonin, is therefore likely to cause the impairments in planning found in depression (19) and during the manic phase of mania (20), although additional factors may contribute to executive dysfunction during euthymia.

Integrating these findings, it may be that serotonin acts on emotion perception systems and influences cognition in a bottom-up fashion (5,12), whereas mood disrupts more complex cognitive processes in higher cortical regions (such as lateral prefrontal cortex [LPFC]) (2) through a more top-down route. This is broadly consistent with recent models of emotion processing that posit the presence of ventral and dorsal streams of affective processing (9) and merits further research. Resistance to these pressures may, furthermore, contribute toward resilience to affective disorders.

As a caveat, these findings may be specific to the tasks studied. Cognitive processes that require the integration of executive processing with emotional processing may be influenced by both mood and 5-HT (which would explain recent findings in which mood state and 5-HT interact to bias cognition) (17), and simple “cold” tasks, which do not require higher prefrontal processing, may be unaffected by both mood and ATD. A further caveat is that we varied 5-HT within subjects but mood between subjects. Future research should vary mood induction within subjects to remove the potential confound of within- versus between-subject effects on task performance.

### Conclusions

In summary, we demonstrate a double dissociation in the influence of 5-HT and mood on cognition and therefore suggest that mood and 5-HT are not closely linked in healthy individuals. While both manipulations influence affect, it may be that ATD mediates emotions and influences “hot” cognition via a bottom-up mechanism, whereas mood influences “cold” cognition via a top-down mechanism. This framework may help us to understand the influence of affect on cognition and hence the changes in cognition seen in affective disorders.

## Figures and Tables

**Table 1:** Group Demographic and Trait Characteristics

Measure	Negative (SD)	Positive (SD)	Neutral (SD)	*F*	*p*
Age	26.1 (6.5)	23.7 (5.0)	22.5 (3.0)	1.41	.26
BDI	3.5 (3.0)	4.4 (2.5)	6.7 (4.4)	2.68	.08
BIS	19.5 (3.3)	19.9 (2.7)	19.3 (3.8)	.11	.90
BAS	39.0 (4.4)	37.2 (7.4)	38.5 (5.5)	.28	.76
IVE-Implusiveness	7.0 (4.9)	6.9 (3.1)	8.8 (4.4)	.72	.49
IVE-Venturesomeness	9.7 (4.7)	10.0 (4.4)	10.5 (4.3)	.08	.93
IVE-Empathy	11.1 (4.3)	13.7 (2.3)	13.0 (3.0)	1.87	.17
Barrat Impulsiveness Scale	63.8 (10.6)	60.6 (10.7)	64.5 (13.3)	.35	.70

ANOVA reveals the groups to be matched (*F/p*). ANOVA, analysis of variance; BDI, Beck Depression Inventory-II; BIS, behavioral inhibition system score; BAS, behavioral activation system score; IVE, Impulsiveness Venturesomeness Empathy questionnaire.

**Table 2:** Affective Go/No-Go

	Happy Words	Sad Words
Female		
BAL	4.40 (.81)	2.80 (.88)
ATD	2.60 (.74)	3.67 (.73)
Male		
BAL	3.19 (.78)	3.94 (.85)
ATD	3.63 (.72)	3.25 (.71)

Distracter errors on the affective go/no-go within each condition (acute tryptophan depletion [ATD] vs. placebo [BAL]/happy vs. sad words) for both male and female subjects (SEM). ATD, acute tryptophan depletion; BAL, nutritionally balanced placebo.
