# Supplementary material for: A Double Dissociation in the Roles of Serotonin and Mood in Healthy Subjects
Source: Biol Psychiatry. 2009 Jan 1;65(1):89–92. doi: 10.1016/j.biopsych.2008.10.001 (PMC2602857; doi:10.1016/j.biopsych.2008.10.001)
Supplement: Supplement 1 [file mmc1.doc]

**Supplementary Material**

**(A) Subject Recruitment**

Subject responded to an advert requesting volunteers that were “*physically healthy, aged 20-50 and have never suffered from depression*.” They were then screened via a structured exclusion interview. Exclusion criteria included past or present history of psychiatric or neurological disorder, past or present psychiatric medication use, past or present DSM-IV depression symptoms, suicide attempts or substance abuse. Subjects with first degree relatives with psychiatric disorders, psychiatric medication use or suicide attempts were also excluded.

**(B) Neurocognitive Tasks**

The affective go/no-go was performed first followed by the one touch tower. This fixed task order replicated that of previous research (1) to maximize across-study comparability.

***Affective Go/No-go***

In the affective go/no-go (AGNG) task (2) a series of words was presented very quickly. Half of the words were targets and half distracters. Subjects were instructed to respond to targets by pressing the spacebar as quickly as possible whilst withholding responses to distracters. In each block (10 total), half the words were ‘happy’ words and half were ‘sad’ words. Prior to each block either the happy or sad words were specified as targets. Every two blocks the target swapped.

Word valence (happy/sad) and treatment (ATD/placebo) were included as within-subjects factors in main effects analysis. Mood group (positive/negative/neutral) was initially included as the between subjects factor but was removed when it was found to not interact with the within subjects factors. Gender (male/female) was then included as the between subjects factor (collapsed across the mood groups) and shown to interact with the two within subjects factors. Simple effects analysis of this interaction then revealed the female specific effect presented in the main manuscript.

*Additional analysis*

There was no interaction between treatment, word valence and gender in either the reaction time (F_1,14_ = 0.14, p = 0.72) or the number of target (omission) errors (F_1,14_ = 0.09, p = 0.77) in female subjects.

***One Touch Tower of London***

In the one touch Tower of London task (OTT) (3) two arrays of three colored balls were presented on the screen, arranged in hanging pockets. Subjects were required to work-out the minimum number of moves required to make the bottom pattern look like the top pattern. They then selected the appropriate number of moves from a list on the bottom of the screen. Trials varied in difficulty, with problems ranging from one to six moves.

Difficulty (1/2/3/4/5/6) and treatment (ATD/placebo) were included as within subjects factors and mood group (positive/negative/neutral) was included as the between subjects factor in main effects analysis.

*Additional analysis*

There was no interaction between mood state and difficulty in the time it took subjects to respond (F_(10,50)_= 1.4, p = 1.9), the total time it took subjects to complete each problem (F_(10,38)_= 1.7, p = 0.083) or in the number of problems that were solved first time (F_(2,28)_= 0.08, p = 0.9).

**(C) Amino-acid Mixtures**

Amino-acid mixtures (prepared by SHS international; Liverpool, UK ) were identical to previous research (4-6):

L-alanine, 4.1g; L-arginine, 3.7g; L-cystine, 2.0g; Glycine, 2.4g; L-histidine, 2.4g; L-isoleucine, 6g; L-Leucine, 10.1g; L-lysine, 6.7g; L-Methionine, 2.3g; L-proline, 9.2g; L-phenylalanine, 4.3g; L-serine, 5.2g; L-threonine, 4.9g; L-tyrosine, 5.2g; L-valine, 6.7g + L-tryptophan, 3.0g—total BAL: 78.2 / TRP-: 75.2g

There was a 20% reduction in quantity for females to take into account lower body-weight. The drinks were prepared by stirring the mixture into approximately 200ml tap water with either lemon-lime or grapefruit flavoring. Subjects reported no side effects apart from transient nausea following ingestion of the drink.

Blood (venous) samples (10ml) were taken immediately before ingestion of the amino-acid drink (T_0_) and approximately 5 hours after administration (T_1_), to determine the crucial TRP/sum of the long neutral amino acids (ΣLNAA) ratio.

**(D) Mood Induction Procedure**

The mood induction procedure (MIP (6)) was programmed in Microsoft Visual Basic 6 (Microsoft Corporation, Redmond, WA, USA) and presented on a Paceblade tablet computer (11” monitor). A set of visual analogue scales (VAS) was administered at T_0_ (admission), T_1_ (5h later pre-MIP) and T_2_ (post-MIP) to determine self-reported mood.

Subjects were presented with 60 (positive, negative or neutral) Velten sentences (7) whilst music was played through Sennheiser HD 202 headphones. Each sentence was presented in the center of the screen for 12s until a ‘next’ button appeared and subjects were able to move on to the next sentence by pressing the space bar. Subjects were instructed to ‘relate the situation described by the sentence to situations in their own lives’, to get ‘as deeply as possible into any mood evoked’ and to ‘feel free to outwardly express any mood evoked.’

The negative version of the MIP contained light grey text on a dark blue background. The music played was either *Adagio for strings, Op. 11* by Samuel Barber or *Adagio in G Mino*r by Tomaso Albinoni. Music was selected by asking the subjects (2 hours prior to testing) which piece was the ‘saddest.’ The positive version featured peach text on a light yellow background. Either *Piano Concerto No. 4, Op. 58 in G Major: III. Rondo: Vivace* by Ludwig van Beethoven or *Serenade No. 13 KV 525 G-Major: I. Serenade. Allegro* by Wolfgang Amadeus Mozart was played. The piece was selected by asking subjects which was the ‘happiest.’ The neutral version featured black text on a white background and *The Planets, Op. 32: VII. Neptune, the Mystic* by Gustav Holst was played.

*Additional analysis*

Main effects analysis is presented within the main article. Within-mood group comparison between T_1_ and T_2_ showed a significant shift towards the ‘sad’ end of the VAS subscale following the negative MIP (T_25_= 2.9, p = 0.008). These data indicate that the negative mood induction procedure was successful. There was a shift towards the positive end of the mood scale following the positive MIP which, although it was not significant (T_25_ = 2.9, p = 0.35), caused a significant difference between the T_2_ ratings in the positive and negative groups (T_25_ = 3.1, p = 0.004) which was not present at T_1_ (T_25_ = 0.9, p = 0.4) or between the negative and neutral groups at T_2_ (T_25_ = 3.2, p = 0.25). There was no shift in ratings following neutral MIP (T_25_ = 1.9, p = 0.24).

**References**

1. Murphy FC, Smith KA, Cowen PJ, Robbins TW, Sahakian BJ (2002): The effects of tryptophan depletion on cognitive and affective processing in healthy volunteers. *Psychopharmacology*. 163:42-53.

2. Murphy FC, Sahakian BJ, Rubinsztein JS, Michael A, Rogers RD, Robbins TW, *et al.* (2000): Emotional bias and inhibitory control processes in mania and depression. *Psychological Medicine*. 29:1307-1321.

3. Owen AM, Sahakian BJ, Hodges JR, Summers BA, Polkey CE, Robbins TW (1995): Dopamine-dependent frontostriatal planning deficits in early Parkinson’s disease. *Neuropsychology*. 9:126-140.

4. Roiser JP, Blackwell AD, Cools R, Clark L, Rubinsztein DC, Robbins TW, *et al.* (2006): Serotonin Transporter Polymorphism Mediates Vulnerability to Loss of Incentive Motivation Following Acute Tryptophan Depletion. *Neuropsychopharmacology*. 31:2264-2272.

5. Cools R, Robinson OJ, Sahakian B (2007): Acute Tryptophan Depletion in Healthy Volunteers Enhances Punishment Prediction but Does not Affect Reward Prediction. *Neuropsychopharmacology*. 33:2291-2299.

6. Robinson OJ, Sahakian BJ (2008): P.2.30 Triple dissociation of serotonergic influence on motivational behaviour under positive, negative and neutral mood. *European Neuropsychopharmacology*. 18:s60-s61.

7. Velten E (1968): A laboratory task for induction of mood states. *Behavioural Research and Therapy*. 6:473-482.
